# The effect of spatial distance between objects on categorization level

**DOI:** 10.3758/s13423-021-01949-2

**Published:** 2021-08-02

**Authors:** Iris K. Schneider, André Mattes

**Affiliations:** 1 Social and Economic Cognition III, University of Cologne, Richard-Strauss-Str. 2, 50931 Cologne, Germany; 2 Individual Differences and Psychological Assessment, University of Cologne, Pohligstr. 1, Cologne, 50969 Germany

**Keywords:** Spatial distance, Categorization, Spatial grouping, Spatial processes, Clumpiness principle

## Abstract

We show that spatial distance between two objects influences how people categorize these objects. We report three (two pre-registered) experiments that show that when objects are presented close together (proximal), they are more likely to be categorized in a superordinate category than when they are presented further apart (distant). In Experiments 1A and 1B, participants provided spontaneous category labels in an open response format. In Experiment 2, we asked participants to indicate their preference for either of two category labels. We found that when objects were close together, they were categorized more often into superordinate categories than when objects were far apart (Experiments 1A and 2). Our findings demonstrate that the categorization of objects is, in part, determined by where they are in relation to other objects.

**Supplementary Information:**

The online version contains supplementary material available at 10.3758/s13423-021-01949-2.

## Introduction

Understanding what objects are is a core task in human cognition. Such categorizations are not made in a vacuum but instead are influenced by variables outside the object (Loken, 2006; Murphy, 2004), such as a person's current needs (Bruner, 1957),emotional state (Isen & Daubman, 1984), or goals (Loken, 2006). One variable that has been mostly overlooked is the spatial distance between objects. Given that two objects cannot occupy the same space at the same time, objects are always somewhere relative to each other in space. Indeed, in everyday life, objects are often presented spatially; for instance, in brochures, online shops, and survey forms. Previous research has shown that spatial distance between objects matters for similarity judgments and choice: An apple and an orange set close together appear more similar (Casasanto, 2008) and might prove a more difficult choice than an apple and an orange set far apart (Lakens, Schneider, Jostmann, & Schubert, 2011; Schneider et al., 2020). Here we go beyond previous work and examine whether *what* people think about objects is influenced by the distance between them. Specifically, we examine whether spatial distance influences categorization levels. Specifically, we propose that people are more likely to think about “fruit” when objects are close together compared to when they are far apart.

In general, objects can be categorized on three levels, superordinate, basic, and subordinate. An apple can be categorized as FRUIT (superordinate), APPLE (basic), or GRANNY SMITH (subordinate) (Murphy, 2004; Rosch, 1975; Rosch et al., 1976).^1^ The level on which an object is categorized has consequences for the properties and features that people attribute to this object. For instance, objects categorized on the superordinate level are often described in terms of abstract or functional properties. As an example, the category FRUIT would bring to mind that one can eat it and that it is healthy (Medin & Smith, 1984; Murphy, 2004). Objects categorized on the basic level are often described in terms of their parts or visual properties. An APPLE is green, round, and small (Murphy, 2004; Rosch et al., 1976; Tversky & Hemenway, 1984). Subordinate levels have all the basic category information but with additional specifications, such as light-green (Medin & Smith, 1984; Rosch et al., 1976; Tversky & Hemenway, 1984). The basic level of categorization is the default level of categorization. When people encounter new objects, they generally use the basic level of categorization (Murphy & Brownell, 1985; Murphy, 2004). However, objects in the world are not always encountered in isolation. Instead, objects often appear together in some spatial arrangement. For instance, people might see an apple and an orange together in a bowl or set out separately on a buffet. We propose that such differences in spatial distance between objects influence the level of categorization.

## Spatial distance and categorization

Why would spatial distance influence categorization? One reason is that spatial distance is informative about the relationships between objects in the world. This is described by the *factor of proximity*, one of the laws of Gestalt theory. This law states that “*that form of grouping is most natural which involves the smallest interval*” (Wagemans et al., 2012; Wertheimer, 1938). Put simply, proximity (i.e., closeness) is a natural cue people use to group objects in their environment. The factor of proximity is so dominant in human perception that spatial groupings based on this principle are not easily unseen. For instance, most people perceive the following dots ●● ●● as two *pairs of dots* instead of *four dots*. The fact that it takes effort *not* to see the dots as pairs, illustrates the profound impact of spatial proximity in visual grouping. In sum, *close together goes together*.

However, the law of proximity refers to visual grouping only, and makes no predictions about categorization levels. Nevertheless, there is reason to assume that proximity can influence categorization level. Proximity to another object creates the perception that the objects *go together* – as illustrated by the dots above. Thus, proximal objects are more likely be perceived as a single group (“pair of dots”) rather than separate elements (“dots”). If a perceiver wants to make sense of a group, they need a level of categorization that can encompass all elements in the group. Superordinate categories are of a higher level of abstraction, less descriptive of the individual object, but more applicable to objects’ shared properties (Isen & Daubman, 1984; Rosch, 1975; Rosch et al., 1976) than basic categories, and therefore likely more suitable as a category.

Furthermore, in the environment, shared category membership and proximity often co-occur. That is, objects that are close to each other often also belong to the same category. For instance, flowers are often close to other flowers, and rocks are usually close to other rocks. In the human-made environment, this association is also present: books are put with other books, and cups are placed with other cups. Because of this learned association, proximity is a strong cue for shared category membership. This idea is summarized in the *Clumpiness Principle* (Casasanto, 2008). This principle proposes that stimuli in the environment are clumped together. Specifically, “physical closeness encourages construing stimuli as members of the same category” (Casasanto, 2008, p. 1053). So far, the relationship between proximity and categorization has not been tested directly. However, the Clumpiness Principle also posits a direct consequence of this categorization process, namely increased perceptions of similarity. This latter relationship has been demonstrated empirically, providing indirect support for the relationship between proximity and categorization.

First, empirical work has shown that proximity facilitates similarity judgments, while distance facilitates dissimilarity judgments. For instance, when people view two similar squares close together, they are faster to determine that they are indeed similar compared to when they are far apart. Conversely, when people view two dissimilar squares far apart, they are faster to determine that they are dissimilar, compared to when they are close together (Boot & Pecher, 2010). Furthermore, when people see two objects close to each other, they process subsequent sentences about similarity faster compared to when they first see two objects far apart (Guerra & Knoeferle, 2014). Conversely, processing sentences about dissimilarity is faster when people first see two objects far apart compared to seeing two objects close together.

Second, spatial distance also influences how subjectively similar people judge stimuli to be. Specifically, stimuli set close together are judged to be more similar than stimuli set far apart. For instance, when people have to judge how similar two words are in meaning, nouns presented close together on a computer screen are perceived as more similar in meaning than nouns presented far apart (Casasanto, 2008). Similarly, two stick figures drawn close together are believed to have more similar political attitudes than stick figures drawn far apart (Winter & Matlock, 2013).

Yet, spatial closeness does not *always* lead to increased similarity. Sometimes, stimuli that are close together appear less similar than stimulu that are far apart. Whether or not proximity leads to more similarity seems dependent on the type of judgment that people make. When people have to judge conceptual similarity – similarity on an abstract dimension – proximity leads to more similarity (Casasanto, 2008). For instance, when people see images of two tools and are asked how similar they are in *use* – a conceptual judgment – they judge tools presented close together as more similar than tools presented far apart. However, when people are asked how similar the tools are in their *visual appearance* –  a perceptual judgment – they judge tools presented close together as less similar than tools presented far apart (Casasanto, 2008).

The observation that proximity only influences similarity in conceptual similarity judgments (i.e., tool use) has been explained through different underlying processes that comprise the similarity judgment (Casasanto, 2008). Specifically, when people have to judge judgment on visual similarity, they can make direct use of the concrete sensory properties of the stimuli. This is because the stimulus itself holds information directly relevant to the task – for instance, through directly observable properties, such as color, shape, texture. In these instances, people do not need to rely on heuristics such as natural co-occurrences. However, when judging similarity on a conceptual level, along abstract dimensions like use or meaning, there is no information in the stimulus that can potentially inform the judgment. In this case, people draw on heuristics that might help them judge similarity. Shared category membership is such a heuristic that can inform similarity judgments. Thus, according to the Clumpiness Principle, proximity leads to similarity for conceptual judgments because of shared category membership (Casasanto, 2008).

## Current research

In the current work we directly examine whether there is a relationship between proximity and categorization as proposed by the Clumpiness Principle. Specifically, we propose that spatial distance between objects influences the degree to which superordinate categorizaties come to mind when viewing objects. Specifically, when objects are close together, people will prefer superordinate categories more than when objects are far apart. While spatial distance between objects has been researched in the domain of similarity judgments and choice, less is known on how it affects categorization. Research on similarity judgments is concerned with whether close spatial distance makes apples and oranges *more similar*. In the current research, we are interested in whether spatial distance makes apples and oranges *more like fruit*. Examining the effect of spatial distance on categorization will further understanding about whether objects take on different meanings depending on spatial distance. Such a finding would be relevant in a range of domains, from methodological considerations about stimulus presentation to decision-making, marketing psychology, and consumer behavior.

We present three experiments. We predict that proximal objects will elicit more superordinate categories than distant objects. Note that we do not focus on subordinate categories in this work because they are very similar to basic categories. We report how we determined our sample size, all data exclusions (if any), all manipulations, and all measures in the experiments. All pre-registrations, data, materials, and analyses scripts can be accessed at: https://osf.io/qzu8x/

## Experiment 1A

Experiment 1A provides a first test of our idea. We showed participants images of objects and ask them to provide a label for each image. We predicted that for close objects, participants would more often name superordinate categories as labels than for far objects.

### Method

#### Power analyses

Because this was the first experiment we ran, we did not do formal power calculations and instead collected 100 participants per condition. Amazon Mechanical Turk provided slightly more participants.

#### Participants and design

A total of 206 participants were recruited from Amazon Mechanical Turk, and paid $0.75 for completing a 7- to 10-min survey that combined the current experiment followed by an unrelated experiment not reported here. A total of 24 participants were excluded from the analyses because they did not provide a response (*N* = 6) or provided multiple labels (e.g., “I see fruit: an apple and an orange”; *N* = 18) on more than one trial. The remaining 182 participants (104 males, 77 females, one unreported) had a mean age of 37.57 years (*SD* = 11.57). Distance (proximal vs. distant) was manipulated between subjects.

#### Procedure

Participants were presented with ten pairs of objects (modelled after Casasanto, 2008). Objects were presented on a white background of 800 × 600 pixels (width × height). Images of the objects were rescaled to fit in a white frame with a width of 150 pixels. We selected the objects based on Rosch (1975) and aimed to use the most typical exemplars for each category. However, the original exemplars were exemplar names, not images. Thus, we selected the exemplars with the highest goodness of fit to the category with the pre-condition that the exemplars would be reasonably different visually. Thus, in cases where we expected the visual representations of the exemplars to lack clear differences, we opted for the next best exemplar. For instance, for the category VEHICLES, the highest rated exemplar was *automobile*, the second rated exemplar was *station wagon*. When presented verbally, these are clearly different, but when presenting images of both, participants might not differentiate between the two. Therefore, we opted for the third highest rated exemplar *truck*, which would have a very different image than automobile. This led to the following objects (category given in brackets): orange and apple (fruit), chair and sofa (furniture), automobile and truck (vehicle; truck being ranked third in the goodness-of-exemplar rating), gun and knife (weapon; knife being ranked seventh after pistol, revolver, machine gun, rifle and switchblade), pea and carrot (vegetable), saw and hammer (carpenter’s tools), robin and sparrow (birds), football and baseball (sports), doll and top (toys), pants and shirt (clothing), (see Appendix A).

**Fig. 1:**
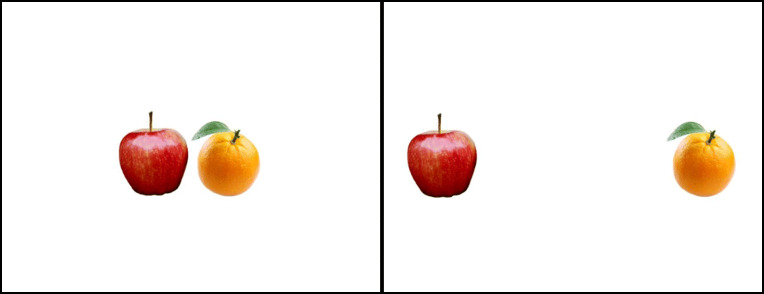
Proximal condition (**left**), with objects close together. Distant condition (**right**), with objects far apart

In the proximal condition, the 150-pixel white frame images were placed adjacent in the center of the white background, with 0 pixels between them. In the distant condition, they were placed 50 pixels from the left and right borders of the background, leaving 444 pixels between them (see Fig. 1). Note that because the images of the objects were placed in a white frame for consistency purposes, the objects themselves do not touch each other in the proximal condition. However, the invisible white frames were indeed placed adjacent.

For half of the participants the object pairs were presented proximal, for the other half they were presented distant. To keep the instructions as neutral as possible, participants were asked to “*Please label these images.*” Participant’s responses were counted as a superordinate category when the label described both objects (e.g., FRUIT), and as basic category when the label described both objects (e.g., an APPLE and an ORANGE). At the end of the experiment, participants indicated age and gender.

#### Statistical analyses

We computed a mixed effects logistic regression in which we entered distance (contrast-coded: distant = -0.5, proximal = 0.5) as a fixed factor and participants and stimuli as random factors. The binary outcome variable was dummy-coded (categorization: basic = 0, superordinate = 1).^2^ We estimated the mixed model in R (R Core Team, 2017) using the packages *lme4* (Bates, Maechler, Bolker, & Walker, 2015) and *lmerTest* (Kuznetsova et al., 2017).

## Results and discussion

The mixed effects logistic regression showed that the probability of using a superordinate category was higher for proximal stimuli (*M* = 57.12%, *SD* = 44.64) than for distant stimuli (*M* = 46.12%, *SD* = 47.13), *b* ± *SE* = 29.78 ± 1.95, *z* = 15.27, *p* < .001. The variance of the intercept by participants and stimuli was σ^2^ = 744.56, and σ^2^ = 23.61, respectively.

**Fig. 2:**
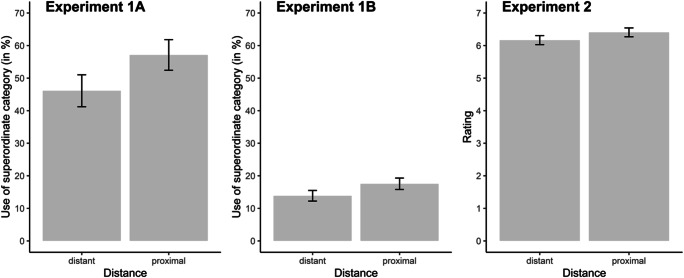
Results of Experiments 1A (**left**), 1B (**center**), and 2 (**right**). Experiments 1A and 1B display the use of superordinate categories (in percentages) for distant and proximal stimulus pairs. Experiment 2 displays the mean rating for distant and proximal stimulus pairs with higher rating scores indicating the tendency towards the superordinate category and lower rating scores indicating the tendency towards the basic category. Error bars represent the standard error of the mean

Thus, in line with our prediction, when objects were close together, people were more likely to use superordinate categories than when objects that were far apart (Fig. 2).

## Experiment 1B

In Experiment 1A, the pattern of the results was in line with our hypothesis. In Experiment 1B we were examined whether the effect would extend to objects that were less prototypical for their category. The pre-registration for this  experiment can be found at: https://osf.io/9qt4m

### Method

#### Power analyses

We assumed an effect size of *d* = 0.24, based on the data collected in Experiment 1A, and aimed for 95% power, resulting in a minimum sample size of 754 participants. In order to fully utilize project resources, we decided to collect a total of 825 participants.

#### Participants and design

Eight-hundred twenty-five participants (426 females, 397 males, two others, *M*_age_ = 38.10 years, *SD* = 11.91) were recruited on Amazon Mechanical Turk and paid $0.40 for 3 min. A total of 23 participants were excluded from the analyses because they did not provide a response (*N* = 3) or provided multiple labels (e.g., “I see fruit: an apple and an orange”; *N* = 18) or both (*N* = 2) on more than one trial. The remaining 802 participants (388 males, 412 females, two other) had a mean age of 38.21 years (*SD* = 11.95). Distance (proximal vs. distant) was manipulated between participants.

#### Procedure

The procedure was identical to Experiment 1A. We used the following objects that were selected from around the median of prototypicality ratings in Rosch (1975; original category given in brackets) that were suitable for visual presentation: lemon and mango (fruit), buffet and lamp (furniture), subway and tractor (vehicle), missile and whip (weapon), potato and green onion (vegetable), crowbar and screws (carpenter's tool), waterskiing and ice skating (sport), fire engine and balloons (toy), tuxedo and stockings (clothing). This resulted in nine object pairs (see Appendix B). The category “birds” was not included in the experiment because in Experiment 1A most of the participants were unable to name the different kinds of birds based on visual depictions, leading to responses such as “a bird and a bird.” The stimulus images were created as in in Experiment 1A. However, because of presentation restrictions in Qualtrics survey software, the background image was resized to 770 × 577 pixels (instead of 800 × 600 pixels as in Experiment 1A). For the proximal condition, stimuli were still placed adjacent, with 0 pixels between them. For the distant condition the images were placed adjacent to the left and right borders (instead of 50 pixels away from them like in Experiment 1A) with 470 pixels between them.

#### Statistical analyses

The statistical analyses were identical to those in Experiment 1A. Following the reviewers’ suggestions, we computed a mixed-effects logistic regression in which we entered distance (contrast-coded: distant = -0.5, proximal = 0.5) as a fixed factor and participants and stimuli as random factors. The binary outcome variable was dummy-coded (categorization: basic = 0, superordinate = 1). The analyses that were planned in the pre-registration are presented in the Online Supplementary Materials.

### Results and discussion

Although there was a tendency to use more superordinate categories for proximal stimuli (*M* = 17.55%, *SD* = 35.04) than for distant stimuli (*M* = 13.86%, *SD* = 32.68), the mixed effects logistic regression did not show a statistically significant effect, *b* ± *SE* = 0.36 ± 0.64, *z* = 0.57, *p* = .572 (Fig. 2). The variance of the intercept by participants and stimuli was σ^2^ = 265.55, and σ^2^ = 0.06, respectively.

Descriptively, participants generally preferred basic categories (*M* = 84.29%, *SD* = 33.91) over superordinate categories (*M* = 15.71%, *SD* = 33.91). To test whether this basic category advantage was also statistically significant, we computed a mixed-effects logistic regression without distance as a fixed-effects factor. This null-model predicts the probability of giving a basic versus a superordinate category regardless of the distance between the stimuli but still taking variability in participants and stimuli into account. This analysis showed that the basic category advantage was statistically significant, *b* ± *SE* = -11.16 ± 0.43, *z* = -24.97, *p* < .001 (participants: σ^2^ = 266.85; stimuli: σ^2^ = 0.06). This suggest that due to the medium levels of typicality of the stimuli (Rosch, 1975), participants were biased towards basic categories, preventing the distance effect found in Experiment 1A from emerging.

## Experiment 2

Experiment 1A showed that in spontaneous labeling tasks people generated on average more superordinate categories for close objects than they did for far objects. However, in Experiment 1B we did not find statistical differences in categorization level between the close and far objects pairs. One reason for this might be that our primary dependent variable in Experiment 1A and 1B was dichotomous. This means that subtle differences might have been harder to detect. For instance, it might be that when two objects are far, the basic category is preferred strongly over the superordinate category, while in the close condition, the basic category is only slightly preferred. This still constitutes a relevant change in preference, but it cannot be detected in a dichotomous choice variable. In Experiment 2 we remedy this by presenting participants with a relative preference scale for either basic category labels or superordinate category labels.

Furthermore, in Experiment 1B it might have been difficult for participants to generate a superordinate category for the pairs, as evidenced by the strong bias towards basic categories compared to Experiment 1A. To make sure that in Experiment 2 our object pairs elicited relatively homogenous category associations across participants, we conducted a pretest to select object pairs.

### Method

#### Power analyses

Based on previous work we aimed to detect an effect of *d*_*z*_ = 0.16 with a power of 95%, which resulted in 309 participants. To account for possible dropout, we set our sample size to 350 participants. The experiment was pre-registered here: https://osf.io/zcmsn/

#### Participants and design

We recruited 350 participants (176 females, 174 males) on Amazon Mechanical Turk and paid $0.30 for 3 min. In line with the pre-registration, six participants were excluded because they were not native English speakers (including them did not change the results), resulting in a final sample of 344 participants (174 females, 170 males, *M*_age_ = 38.59 years, *SD* = 11.88). Because we used a scale ranging from preference for basic to superordinate category, it was not possible for participants to give multiple labels like in Experiment 1A and 1B. Nor was it possible for participants to skip trials. Therefore, in this data set, there were no missing responses and no exclusions. Spatial distance was manipulated within participants (proximal vs. distant; see below for details on how we realized this). The stimulus/distance combination was counterbalanced between participants, such that the objects that were presented distant in one counterbalance condition, were presented proximal in the other condition and vice versa. Participants completed seven proximal and seven distant trials.

#### Procedure

**Fig. 3:**
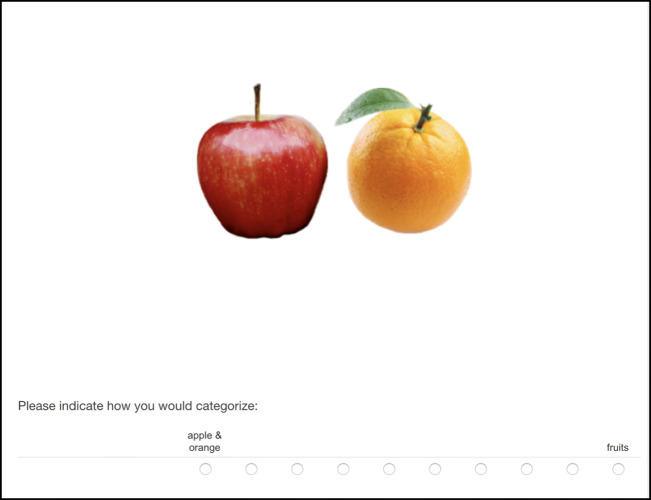
Example of a proximal pair in Experiment 2

Participants gave their informed consent and were subsequently presented with 14 pairs of objects (see Appendix C), half presented far apart and half presented close together (modelled after Casasanto, 2008). We asked participants to “*Please indicate how you would categorize*” on a 10-point scale. The scale was anchored on the left with basic categories (e.g., “apple & orange” = 1), and on the right with a superordinate category (e.g., "fruits" = 10) (see Fig. 3). Thus, higher scores indicated a stronger preference for the superordinate category for a given pair.

In order to select appropiate stimuli, we first generated 35 pairs of objects for our pretest (see Online Supplemental Material); eight of these pairs were the exact same pairs as used in Experiment 1A. We then selected from this initial pool those objects that elicited the most agreement over participants both on the basic and on the superordinate categories (see Online Supplemental Material for extensive descriptions and ratings). This resulted in the 14 object pairs for each of which participants indicated on a scale how they would categorize them. Notably, the pretest results also led to the inclusion of the eight pairs from Experiment 1A, suggesting that these pairs elicited strong agreement among participants for both the superordinate and basic categories associated with the objects (see Appendix C).

Participants indicated for each pair how they would categorize it on a 10-point scale ranging from the basic category to the superordinate category. The scale ends were: pants^3^ & shirt – clothes; burger & French fries – fast food; fork & knife – silverware; dollar bill & quarter – money; apple & orange – fruits; couch & chair – furniture; necklace & ring – jewelry; carrot & peas – vegetables; cat & dog – pets; car & truck – vehicles; gummy bears & chocolate – candy; hammer & saw – tools; doll & top – toys; gun & knife – weapons (see Online Supplemental Material for pre-test). At the end of the survey, participants indicated age, sex, and whether they were native English speakers.

Objects were presented on a white background measuring 800 × 600 pixels (width × height). We rescaled object images to a maximum width of 200 pixels and a maximum height of 200 pixels without changing the width-to-height ratio. We ensured that both objects were equally large by requiring the circumference of both object images to be equal. In the proximal condition, images were presented in the center of the background with 2 pixels between them. In the distant condition images were presented 2 pixels from the left and right edge of the background. Thus, in the distant condition, images were always separated by at least 396 pixels (mean distance = 492 pixels, SD = 90 pixels, min = 409 pixels (pets), max = 752 pixels (knife and fork, portrait orientation), second max = 578 (pants and shirt)). Finally, to fit the Qualtrics Survey software, images (objects and background) were rescaled to 740 × 555 pixels.

#### Statistical analyses

As in Experiments 1A and 1B, we followed the reviewers' suggestions and conducted a mixed-model analysis. We report our pre-registered analyses in the Online Supplementary Material. The model specification was as in the previous two experiments, except that due to the continuous nature of the outcome variable (rating scale), we employed a mixed effects linear regression (in contrast to the mixed effects logistic regression in Experiments 1A and 1B). Distance was entered as a contrast-coded fixed factor (distant = -0.5, proximal = 0.5), participants and stimuli were entered as random factors.

### Results and discussion

The mixed-model analysis showed that superordinate categories were preferred more for proximal objects (*M* = 6.41, *SD* = 2.52) than for distant objects (*M* = 6.17, *SD* = 2.58), *b ± SE* = 0.24 ± 0.08, *t*(4,458.00) = 2.83, *p* = .004 (Fig. 2). The variance of the intercept by participants and stimuli was σ^2^ = 5.08, and σ^2^ = 1.37, respectively. For the scale ends “dollar bill & quarter – money,” we erroneously presented a Euro bill and Euro coin. However, excluding this stimulus pair did not change the pattern of results, *b ± SE* = 0.26 ± 0.09, *t*(4,120.43) = 2.91, *p* = .004 (variance of the intercept by participants: σ^2^ = 5.41; by stimuli: σ^2^ = 1.00). This means that when objects were close together, people prefered the superordinate level of categorization more than when objects were far apart.

The findings from Experiment 2 provide a conceptual replication of our findings in Experiment 1A, using a different dependent variable and extending the number of stimuli. Notably, eight of the stimulus pairs that emerged as most suitable from extensive pretests turned out to be the exact pairs from as in Experiment 1A, suggesting that for spatial distance to influence categorization, the objects need to have strong associations with both the basic as well as the superordinate categories.

## General discussion

We examined whether the spatial distance between objects in the world influences categorization levels. We hypothesized that objects presented close together would be categorized more often in superordinate categories than objects presented far apart. Experiment 1A and Experiment 2 show that this is the case. Participants preferred superordinate over basic categories when presented with the two categorization levels, and also generated more superordinate categories in an open response format. This suggests that spatial distance between objects not only influences subjective ratings, but also what comes to people’s minds spontaneously.

Experiment 1B painted a different picture. In this experiment we used objects that were only moderately prototypical for the superordinate category from which we selected them. We found that almost all responses – regardless of distance – referred to basic categories, suggesting that a superordinate category did not come to mind, or at least less strongly than the basic categories. Perhaps this means that the findings from Experiments 1A and 2 are limited to objects that have relatively clear basic and superordinate categories associated with them. Experiment 2 seems to indirectly support this reasoning. The objects used in Experiment 2 were carefully pretested to elicit homogenous basic and superordinate categories across all participants. Using these stimuli, the effect of distance on preferences for superordinate categories emerged. Notably, the pretest for Experiment 2 also showed, in retrospect, that the stimuli we used for Experiment 1A elicited these homogenous responses as well, suggesting that prototypicality or category fit might be an important moderator of the effect of spatial distance on categorization level.

Taken together, these findings suggest that, at least for prototypical exemplars, spatial distance influences the categorization level. This finding is in line with the Clumpiness Principle (Casasanto, 2008), which suggests that proximity, category membership, and similarity are interrelated in human cognition. However, hitherto, the empirical evidence for this principle was limited to the relationship between proximity and similarity (Boot & Pecher, 2010; Casasanto, 2008; Guerra & Knoeferle, 2014; Winter & Matlock, 2013), while the link between proximity and category membership lacked empirical evidence. In this work we provide this evidence and show that – at least for prototypical exemplars – proximity increases the degree to which objects are categorized together in a superordinate category.

Our work also demonstrates a moderation of the basic category level advantage. In general, people prefer the basic category and this level of categorization is the default level of categorization when people encounter objects (see Online Supplemental Materials for analyses of this in our data). When people have to categorize an object, they are faster to determine whether something is an APPLE, rather than FRUIT (Fan et al., 2012; Johnson & Mervis, 1997; Murphy, 2004; Murphy & Smith, 1982; Rosch et al., 1976). While the basic category advantage is a robust phenomenon, different moderators have been documented (e.g., Macé et al., 2009; Murphy & Brownell, 1985; Rogers & Patterson, 2007). We add to this work by identifying a contextual moderator, namely spatial distance between objects. Our findings show that when (prototypical) objects are close together, superordinate categories are preferred more (and thus basic categories less) than when they are far apart.

### Future research

In all our studies, we used self-report measures to examine categorization levels. Using these measures, we found that people preferred superordinate categories more for proximal than for distant stimuli. At the same time, we might not have been able to detect more subtle effects. For instance, in Experiment 1B, people named more basic categories than in Experiment 1A. Above, we have suggested that this might be because spatial distance does not influence categorization for objects that are less prototypical. However, there might be another possibility. Perhaps in Experiment 1B, proximity did suggest shared category membership, but finding a suitable superordinate category *label* might have been too difficult. Eventually, participants might have given up, reverting to the basic category instead. Our self-reports would not have been able to pick up on such differences in difficulty, but more process-oriented measures, such as response times or mouse-tracking, would. One could for instance examine whether categorization of proximal pairs, regardless of category labels, take longer than those for distant pairs. Using such measures to get more fine-grained insight into the effects reported here would be a fruitful avenue for future work.

Our work adds to existing work examining the effect of spatial distance between objects on judgment processes. In particular, previous work has shown that proximity influences similarity judgments (Casasanto, 2008; Winter & Matlock, 2013) as well as difficulty in dichotomous choice (Lakens et al., 2011; Schneider et al., 2020). One question is how these different processes unfold temporally. One possible sequence could be that upon perceiving objects, the distance between objects influences categorization levels in line with the Clumpiness Principle (Casasanto, 2008). Next, this shared category membership influences similarity because members of the same category are more similar than members of different categories (Brewer & Weber, 1994; Brown, Novick, Lord, & Richards, 1992; Mussweiler, 2003; Mussweiler & Bodenhausen, 2002). Finally, choosing between more similar options is more difficult than choosing between dissimilar choice options (Mellers & Biagini, 1994). Testing these processes in a single model is an exciting direction for future research.

Finally, in the work presented here we found that superordinate categories were preferred more for proximal than for distance stimuli, in line with our predictions. One might argue that the opposite prediction is also possible: proximal stimuli lead to less preference for superordinate categories. For instance, in similarity judgments, close spatial distance leads to more similarity when the judgment task is conceptual, but less similarity when the judgment task is focused on visual comparison (Casasanto, 2008). Could a similar reversal be observed for categorization tasks? Our data cannot speak to this question. However, future research might juxtapose visual and conceptual categorization to investigate this question.

### Conclusions

In this work, we examined the influence of spatial distance between objects on categorization level. We found that when objects are set close together, people prefer superordinate categorization more than when the objects are set far apart. As such, our work shows that spatial distance between objects systematically influences the categories that come to people’s minds and the meaning of the objects they encounter. An apple and an orange set far apart, are just that – “an apple and an orange.” But when the same apple and orange are set close together, they become “fruit.” In sum, *what* an object is, is in part determined by *where* it is relative to other objects.

### Supplementary Information

ESM 1 (DOCX 40.1 kb)

## Stimulus pairs in Experiment 1B

### Appendix C: Stimulus pairs in Experiment 2

(proximal condition only, see Online Supplemental Materials for complete stimulus sets)

## Footnotes

1 What is considered a superordinate, basic, or subordinate category is relatively stable. However, training and experience might shift the basic category to higher or lower levels. For instance, for people who have no experience with trees, the basic level would be TREES, while people who have lived among trees all their life, the basic category might constitute ELM or OAK and TREES would be the superordinate level (G.L. Murphy, 2004).

2 We initially planned and pre-registered to aggregate the data within participants and across stimuli to conduct a *t*-test. However, the reviewers suggested running a mixed model, properly taking the variability in stimuli into account. We report the results of our initial analyses in the Online Supplementary Material.

3 “Pants” is the most appropriate noun for American English-speaking samples. For British English samples, “trousers” would have been more appropriate.
