# Supplementary material for: The effect of spatial distance between objects on categorization level
Source: Psychon Bull Rev. 2021 Aug 2;29(1):230–42. doi: 10.3758/s13423-021-01949-2 (PMC8858281; doi:10.3758/s13423-021-01949-2)
Supplement: Supplementary file 1 — (DOCX 40.1 kb) [file 13423_2021_1949_MOESM1_ESM.docx]

**Supplemental Materials**

We initially planned to perform the statistical analyses on data that was aggregated within participants (and across stimuli). However, the reviewers suggested to run mixed model analyses which – unlike the aggregation approach – would take the variability between stimuli into account. For the sake of transparency, we here report the results of our initial analyses which are mostly similar to the results reported in the main document.

**Experiment 1A**

We aggregated the data by computing the frequency of superordinate categories each participant provided. A visual inspection of the frequency distribution revealed that the data deviated from a normal distribution. We thus opted for the Mann-Whitney-U-test, the non-parametric equivalent of an independent *t*-test, to analyze the data. For each comparison, we report the effect size *d* that we derived from the *Z*-value of the Mann-Whitney-U-test (Fritz, Morris, & Richler, 2012) obtained by the *coin* package for R (Hothorn, Hornik, Wiel, & Zeileis, 2008).

Participants who saw objects close together used superordinate categories more (*M* = 5.70, *SD* = 4.46, mean rank = 98.50) than those who saw objects far apart (*M* = 4.55, *SD* = 4.67, mean rank = 84.65), *U*= 4,770, *Z* = 1.87, *p* = .031, *d* = 0.28 (one-sided).

**Experiment 1B**

As in Experiment 1A, we aggregated the data by computing the frequency of superordinate categories each participant provided. Visual inspection of the distribution showed that the data deviated from a normal distribution. Thus, we used the non-parametrical equivalent of an independent *t*-test, the Mann-Whitney-U-test. In line with our hypothesis, participants who saw the objects proximal used more superordinate categories (*M* = 1.57, *SD* = 3.14, mean rank = 414.78) than those who saw the objects distant (*M* = 1.24, *SD* = 2.93, mean rank = 388.22), *U* = 85,727, *Z* = 2.29, *p* = .011, *d* = 0.16 (one-sided).

While the expected effects emerged in the non-parametric test, the pattern of results was less clear in parametric statistical tools. It is important to keep in mind that the results of parametric tests need to be considered very carefully when the assumptions regarding the distribution are violated as is the case here. For the sake of completeness, we report the analysis nonetheless. We pre-registered a repeated measures ANOVA with type of categories (superordinate vs. basic) as within-subjects factor and distance (proximal vs. distant) as between-subjects factor. In the pre-registration, we detailed that we expected an interaction effect such that more basic categories would be used for distant stimulus pairs while more superordinate categories would be used for proximal stimulus pairs. Descriptively speaking, the predicted pattern emerged. Participants who saw the objects proximal used more superordinate categories (*M* = 1.57, *SD* = 3.14) than those who saw the objects distant (*M* = 1.24, *SD* = 2.93), while participants who saw the objects distant used more basic categories (*M* = 7.71, *SD* = 2.93) than participants who saw the objects proximal (*M* = 7.37, *SD* = 3.14). However, the interaction did not reach the level of statistical significance, *F*(1, 800) = 2.41, *p* = .121, η*_p_*^2^ < .01. There was however, a significant main effect for the level of categorization, *F*(1, 800) = 816.84, *p* < .001, η*_p_*^2^ = 0.51. Basic categories were used more often (*M* = 7.54, *SD* = 3.04) than superordinate categories (*M* = 1.41, *SD* = 3.04) regardless of distance (basic category advantage). Again, note that these are not appropriate analyses and the result should be not be overinterpreted.

**Pretests for Experiment 2**

In order to find labels for the anchors of the rating scale in Experiment 1 (i.e. labels for the “superordinate categories” end and labels for the “basic categories” end), we conducted two pretests. The goal of Pretest 1 was to find superordinate categories for two objects presented in a picture. Pretest 2 then identified the corresponding basic categories for the objects.

**Participants and Design**. One hundred participants participated in Pretest 1 (52 males, 48 females, *M*_age_ = 35.72 years, *SD* = 10.88) and one hundred in Pretest 2 (56 males, 44 females, *M*_age_ = 34.75 years, *SD* = 10.50). In Pretest 1, all participants were presented with all object pairs and indicated a label for both objects. In Pretest 2, each participant was presented with only one of the objects of each stimulus pair and indicated a label for that object only.

**Procedure**. We generated a pool of 35 object pairs that shared the same superordinate categories but were different from each other. In Pretest 1, participants were shown pictures of these object pairs and asked to “*indicate which category fits both objects*” in an open response. Object pairs for which the same superordinate category was named more than forty times were selected for Pretest 2. In Pretest 2, the participants were presented with one object of the object pairs and asked to “*please label the object*." After they had given their response, they were asked to indicate "*how typical the objects [was] for the category X*," with *X* being the superordinate category that was identified in Pretest 1. The rating slider ranged from 0 (“*not typical at all*”) to 100 (“*very typical*"). When participants gave a rating of below 20, i.e., they rated the previously identified superordinate categories as untypical, we sked them "*what would be an adequate name for the category*?".

**Results**. In Pretest 1, 28 out of 35 object pairs were given the same superordinate categories at least forty times (100 participants labeled each object pair). In Pretest 2, for 16 object pairs, the individual objects were given the same basic category at least twenty times (50 participants labeled each object). For two of these object pairs (flowers and balls), the corresponding superordinate category was also named more than ten times. As such, for these pairs, the individual objects seemed to bring to mind both the basic category as well as the superordinate category, which would have been a confound in our main experiment, and we did not use these object pairs. The final set of stimuli thus consisted of 14 object pairs. The pretests ensured that they shared the same superordinate category and that each individual object evoked the same basic category across participants.

Finally, the average typicality score was 85.17 (*SD* = 7.84), indicating that the individual objects were generally typical for the selected superordinate category. Additionally, we found that participants named an alternative superordinate category for individual objects only on 0.62 times on average (*SD* = 1.45), emphasizing the fit of the individual objects to our selected superordinate categories. To illustrate this with an example as presented in Table 1, 54 out of 100 participants gave the superordinate category "clothes" for the pants and t-shirt, which were presented together in Pretest 1. In Pretest 2, when presented with an image of the pants only, 33 out of 50 participants gave it the basic category “pants”. Likewise, when presented with an image of the t-shirt only, 40 out of 50 participants gave it the label “t-shirt”. Only two participants gave the superordinate category “clothes” as a label for the pants. These results indicate that the pictures of the pants and t-shirt used in Pretest 1 bring to mind the superordinate category “clothes” and the basic categories "pants" and "t-shirt". Thus, the stimulus pair was selected for Experiment 1.

Table 1

*Results of both pretests: Frequencies of superordinate and basic categories. For example, the first row shows that when presented with two bikes, 81 out of 100 participants said “bikes”. Only two categorized the mountain bike as a “mountain bike” (basic level). Instead, 23 categorized the mountain bike as “bike” (superordinate level). This means that most participants had no trouble naming the superordinate category, most did not have basic categories at the ready for the different objects. Thus, the stimulus pair was not selected for Experiment 1.*

| Superordinate categories  (2 objects shown at a time) | |  | Basic Categories  (1 object shown at a time) | | |
| --- | --- | --- | --- | --- | --- |
| Expected | *Mentioned by*  *N* (of 100) |  | Expected | *Mentioned by*  *N* (of 50) | *Mentioned by*  *N* (superord. cat.)^A^ |
| bikes | 81 |  | mountain bike  Holland bike | 2  0 | 23  25 |
| birds | 97 |  | raven  pigeon | 8  24 | 14  20 |
| bottles | 36 |  | beer bottle  wine bottle | - | - |
| bread | 75 |  | toast  rye bread | 26  0 | 17  38 |
| cloths | 8 |  | cleaning cloths  tissues | - | - |
| clocks | 47 |  | grandfather clock  watch | 15  43 | 26  0 |
| **clothes** | **54** |  | **pants**  **t-shirt** | **33**  **40** | **2**  **0** |
| colors | 35 |  | red  blue | - | - |
| **silverware** | **41** |  | **fork**  **knife** | **42**  **26** | **3**  **2** |
| soda | 62 |  | Coke  Fanta | 5  1 | - |
| **fast food** | **45** |  | **burger**  **French fries** | **21**  **28** | **0**  **0** |
| fish | 93 |  | goldfish  carp | 34  1 | 14  49 |
| flags | 94 |  | Canadian flag  American flag | 19  13 | 27  36 |
| flowers | 97 |  | rose  tulip | 38  20 | 11  23 |
| **fruits** | **95** |  | **apple**  **orange** | **41**  **43** | **8**  **4** |
| **furniture** | **65** |  | **couch**  **chair** | **24**  **45** | **2**  **4** |
| games | 88 |  | board game  cards | 19  32 | 20  0 |
| glasses | 66 |  | wine glas  beer glas | 25  0 | 19  38 |
| craft supplies | 16 |  | scissors  glue | - | - |
| **jewelry** | **90** |  | **necklace**  **ring** | **33**  **33** | **11**  **2** |
| lights | 60 |  | lamp  fairy lights | 37  0 | 1  25 |
| **money** | **71** |  | **dollar bill**  **quarter** | **30**  **34** | **14**  **3** |
| pasta | 77 |  | tortellini  spaghetti | 12  13 | 23  22 |
| pens | 9 |  | pen  pencil | - | - |
| **pets** | **66** |  | **dog**  **cat** | **43**  **47** | **1**  **1** |
| religion | 0 |  | mosque  church | - | - |
| shoes | 84 |  | sneakers  high heels | 12  9 | 17  20 |
| balls | 77 |  | basketball  soccer ball | 37  31 | 11  11 |
| stone | 25 |  | brick  cliff | - | - |
| **candy** | **78** |  | **Gummi bears**  **chocolate** | **31**  **42** | **10**  **5** |
| **tools** | **84** |  | **hammer**  **saw** | **40**  **32** | **7**  **3** |
| **toys** | **89** |  | **doll**  **top** | **46**  **29** | **3**  **5** |
| **vegetables** | **87** |  | **carrot**  **peas** | **41**  **27** | **6**  **3** |
| **vehicles** | **83** |  | **car**  **truck** | **38**  **25** | **5**  **0** |
| **weapons** | **90** |  | **gun**  **knife** | **39**  **31** | **3**  **1** |

*Note*: The expected superordinate category (first column) and the number of times it was named among 100 participants (second column). The expected basic categories (third column) and the number of times they were named among 50 participants each (fourth column). The number of times the expected superordinate category was named when asking for a basic category (fifth column). Objects of superordinate categories that were named less than 40 times in the first pretest were not included in the second pretest, hence the dashes in the last two columns. Categories that were selected for the main experiment are printed in bold.

^A^ Number of times the intended superordinate category was given when asking for a basic category.

**Analyses Experiment 2**

We aggregated the data by computing the mean rating for each participant for proximal and distant stimuli. In line with the directional hypothesis, we performed a one-tailed paired *t*-test and found that superordinate categories were preferred more for proximal objects (*M* = 6.41, *SD* = 2.52) than for distant objects (*M* = 6.17, *SD* = 2.58), *t*(343) = 2.50, *p* = .006, *d* = 0.13, 95% CI [-0.02, 0.28]. For the scale ends “dollar bill & quarter - money”, we erroneously presented a Euro bill and Euro coin. However, excluding this stimulus pair did not change the pattern of results, *t*(343) = 2.55, *p* = .006, *d* = 0.14. This means that when objects are close together, people prefer the superordinate level of categorization more than when objects are far apart.

We further suspected that there were large individual differences in the tendency to prefer basic vs. superordinate categories. This notion is supported by a strong correlation between ratings of distant and proximal stimulus pairs, *r*(342) = .76, *p* < .001. The more the participants rated proximal stimulus pairs in the direction of superordinate categories, the more they also rated distant stimulus pairs in the direction of superordinate categories. Conversely, the more they rated distant stimulus pairs in the direction of the basic categories, the more they also rated proximal stimulus pairs in the direction of basic categories. This means that the participants had a clear tendency to either rate most of the stimulus pairs in the direction of superordinate categories or in the direction of basic categories, regardless of the distance between the stimuli. Despite this tendency, for proximal stimulus pairs participants still favored the superordinate categories in contrast to distant stimulus pairs for which they favored the basic categories.

**Basic Category Advantage**

We examined the basic category advantage in our aggregated data. See below the analyses for each experiment.

**Experiment 1A**

An asymptotic Wilcoxon-Pratt signed-rank test revealed that participants used superordinate categories (*M* = 5.12, *SD* = 4.59) slightly more than basic categories (*M* = 4.76, *SD* = 4.54), *Z* = 2.31, *p* = .021.

**Experiment 1B**

An asymptotic Wilcoxon-Pratt signed-rank test revealed that that participants used basic categories (*M* = 7.54, *SD* = 3.04) significantly more often than superordinate categories (*M* = 1.41, *SD* = 3.04), *Z* = 20.64, *p* < .001.

**References**

Fritz, C. O., Morris, P. E., & Richler, J. J. (2012). Effect size estimates: Current use, calculations, and interpretation. *Journal of Experimental Psychology: General*, *141*(1), 2–18. https://doi.org/10.1037/a0024338

Hothorn, T., Hornik, K., van de Wiel, M. A., & Zeileis, A. (2008). Implementing a Class of Permutation Tests: The **coin** Package. *Journal of Statistical Software*, *28*(8). https://doi.org/10.18637/jss.v028.i08
